# SARS-CoV-2 infection causes prolonged cardiomyocyte swelling and inhibition of HIF1α translocation in an animal model COVID-19

**DOI:** 10.3389/fcvm.2022.964512

**Published:** 2022-10-17

**Authors:** Margo Daems, Laurens Liesenborghs, Robbert Boudewijns, Steven J. Simmonds, Sirima Kraisin, Jore Van Wauwe, Ilona Cuijpers, Jana Raman, Nadèche Geuens, Tina Van Buyten, Marleen Lox, Peter Verhamme, Sophie Van Linthout, Kimberly Martinod, Stephane Heymans, Carsten Tschöpe, Johan Neyts, Elizabeth A. V. Jones

**Affiliations:** ^1^Centre for Molecular and Vascular Biology, KU Leuven, Leuven, Belgium; ^2^Laboratory of Virology and Chemotherapy, Department of Microbiology, Immunology and Transplantation, Rega Institute for Medical Research, KU Leuven, Leuven, Belgium; ^3^Department of Cardiology, CARIM School for Cardiovascular Diseases, Maastricht University, Maastricht, Netherlands; ^4^Virchow Clinic, Berlin Institute of Health Center for Regenerative Therapies (BCRT), Charité - University Medicine Berlin, Berlin, Germany; ^5^German Center for Cardiovascular Research (DZHK), Berlin, Germany; ^6^Department of Cardiology and Pneumology, Charité - University Medicine Berlin, Berlin, Germany

**Keywords:** COVID-19, HIF1α, cardiac oedema, diastolic dysfunction, hypoxia, pericyte loss

## Abstract

Recovered COVID-19 patients often display cardiac dysfunction, even after a mild infection. Most current histological results come from patients that are hospitalized and therefore represent more severe outcomes than most COVID-19 patients face. To overcome this limitation, we investigated the cardiac effects of SARS-CoV-2 infection in a hamster model. SARS-CoV-2 infected hamsters developed diastolic dysfunction after recovering from COVID-19. Histologically, increased cardiomyocyte size was present at the peak of viral load and remained at all time points investigated. As this increase is too rapid for hypertrophic remodeling, we found instead that the heart was oedemic. Moreover, cardiomyocyte swelling is associated with the presence of ischemia. Fibrin-rich microthrombi and pericyte loss were observed at the peak of viral load, resulting in increased HIF1α in cardiomyocytes. Surprisingly, SARS-CoV-2 infection inhibited the translocation of HIF1α to the nucleus both in hamster hearts, in cultured cardiomyocytes, as well as in an epithelial cell line. We propose that the observed diastolic dysfunction is the consequence of cardiac oedema, downstream of microvascular cardiac ischemia. Additionally, our data suggest that inhibition of HIF1α translocation could contribute to an exaggerated response upon SARS-CoV-2 infection.

## Introduction

The long-term consequences of SARS-CoV-2 infection, both after clinically mild or more severe manifestations, are not yet understood. SARS-CoV-2 infection has several implications beyond the traditional respiratory syndrome COVID-19, including the risk of developing severe cardiac injury, which in turn may exacerbate its severity (1–3). In autopsy specimens from patients who died from COVID-19, significant amounts of viral RNA were found in the heart (4). Over 60% of patients hospitalized for COVID-19 present with clear signs of myocardial injury (5), with between 20 and 63% of patients showing increased cardiac damage as evidenced by specific biomarker levels (2, 5, 6). Hospitalized patients present with a range of cardiac dysfunctions, including left ventricular wall motion abnormalities (23.7%), ventricular dysfunction (right ventricle 26.3%, left ventricle 18.4%), diastolic dysfunction (13.2%), and pericardial effusions (7.2%) (5). Additionally, long-term consequences on cardiac function have been suggested, as evidenced by the presence of cardiac fibrosis (7, 8). Moreover, in a large study of 150,000 veterans who recovered from COVID-19, cardiovascular risk remained elevated for at least 12 months, even in people who had not been hospitalized (9). These results make it clear that, at least with respect to earlier strains of SARS-CoV-2, significant cardiovascular risk remains even if the disease progression was not severe. As such, understanding cardiac consequences of milder infections is essential.

Here, we aimed to investigate cardiac effects of SARS-CoV-2 infection to better understand the mode of action of the SARS-CoV-2 virus by using an established hamster model. In this model, hamsters become sick and recover over a period of ~10 days, without any significant mortality of animals being present. Using echocardiography, we demonstrate that SARS-CoV-2 infection induces the development of diastolic dysfunction. We find no evidence of fibrosis in the infected hearts. We found that cardiomyocyte enlargement occurs rapidly after infection. This rapid increase suggested swelling of the cells, which was confirmed by measuring the water content of the hearts. Histologically, we observe signs of microvascular dysfunction; these include a transient presence of fibrin-rich microthrombi at 4 days post infection (dpi), followed by increased vascular permeability at 14 dpi, and a reduced pericyte coverage of capillaries in the heart. This microvascular dysfunction coincides with hypoxia at 4 dpi, as indicated by increased HIF1α expression. Surprisingly, however, SARS-CoV-2 infection prevented the translocation of HIF1α to the nucleus at 4 dpi, preventing the cell from efficiently resolving hypoxia. Our data suggests that the combination of microthrombi and loss of capillary tone causes hypoperfusion and in turn hypoxia in cardiomyocytes. As hypoxia is not resolved efficiently, the heart becomes stiffer and “swollen” as well as showing diastolic dysfunction.

## Method/material

### Animal handling

Experiments were performed according to the European Directive (2010/63/EU) and approved by the Animal Care and Use Committee of KU Leuven (P083/2020). Twelve week old female Syrian hamsters were inoculated intranasally with 2 × 10^6^ TCID_50_ SARS-CoV-2, as validated previously (10), strain BetaCov/Belgium/GHB-03021/2020 (EPI ISL 407976|2020-02-03), recovered from a nasopharyngeal swab from a patient who returned from Wuhan in February 2020, and sacrificed after 4 dpi, 14 dpi, or 35 dpi (11, 12). Studies were limited to female hamsters to mimic a milder disease progression (13).

### SARS-CoV-2 RT-QPCR and end-point titrations

Hamster tissues were homogenized with bead disruption (Precellys) in 350 μL RLT buffer (RNeasy Mini kit, 74004 Qiagen) and RNA was extracted according to the manufacturer's instructions. RT-qPCR was performed on a LightCycler96 platform (Roche) with iTaq Universal Probes One-Step RT-qPCR kit (1725140, BioRad) with N2 primers and probes targeting the nucleocapsid (11). Standards of SARS-CoV-2 cDNA (IDT) were used to quantitatively express viral load as genome copies per mg tissue. For end-point titrations, Vero E6 cells (African green monkey kidney, ATCC CRL-1586) were cultured in minimal essential medium (15188319, Gibco) supplemented with 10% fetal bovine serum, 1% L-glutamine (25030149, Gibco), and 1% bicarbonate (25080094, Gibco). Homogenized lung tissue in minimal essential medium was titrated on confluent Vero E6 cells in 96-well plates. Viral titres were calculated with the Reed and Muench method and expressed as 50% tissue culture infectious dose (TCID_50_) per mg tissue.

### Transthoracic echocardiography

Twelve week old hamsters were anesthetized with an intraperitoneal injection of 100 mg/kg ketamine (Nimatek, Eurovet) and 10 mg/kg xylazine (Xyl-M, V.M.D. nv/sa) (14). Complete transthoracic 2D M-mode echocardiography was performed using a MS 250 transducer (13–24 MHz) connected to a Vevo 2100 echocardiograph (Visual Sonics). Hamsters were placed in a supine position on a heating pad to maintain core body temperature between 37.5 and 37.7°C, measured using a rectal probe. Electrocardiography recordings were performed to monitor heart and breathing rate. If needed, anesthesia dosage was modified to ensure constant heart rates between all hamsters. HR and cardiac dimensions were assessed on the parasternal short-axis M-mode imaging. CO, EF, LV mass, and FS were calculated based on parasternal short-axis M-mode recordings. Left ventricular filling was assessed by pulsed wave Doppler trans-mitral flow velocity tracings, including peak E and A wave velocities, MV DT, and IVRT. Myocardial E' and A' diastolic peak velocity was measured by tissue Doppler imaging at the lateral mitral annulus. E/A, E/A', E/E', and E'/A' ratios were calculated. At least three stable cardiac cycles were averaged for all measurements. More details are available in the online Supplementary material.

### Organ collection

Twelve week old hamsters were sacrificed at 4 dpi, or after echocardiography either at 14 dpi or 35 dpi. Hamsters were euthanized by excision of the heart under anesthesia. Approximately 5–30 mg of the apex of the heart was placed in RTK buffer for RNA extraction, and the remaining was fixed overnight in 4% paraformaldehyde (PFA) at room temperature. For the perfusion experiment, hamsters were injected intracardially with 7.2 μl/g of bodyweight Lycopersicon Esculentum (Tomato) Lectin (B-1175, Vector labs), which was allowed to circulate for 1 min. Sacrifice proceeded as described above. For wet weight vs. dry weight measurements, part of the left ventricle was weighed during dissection. The piece of ventricle was placed at 70°C and dry weight was measured after 72 h.

### Histological analysis

All antibodies and concentrations are listed in Supplementary Table 1.

Four μm paraffin sections were cut and deparaffinized. For laminin staining, proteinase K epitope retrieval was used (1/500 in PBS, 3115828001, Sigma Aldrich). For NG2 and HIF1α staining, citrate buffer (0.1M Target Retrieval Solution, DAKO S1699, Agilent) was used. Pericyte coverage was determined by counting the number of capillary vessels (isolectin B4+) that are covered by NG2+ cells. Capillary vessels are defined as vessels smaller than 10 μm in diameter. For vWF staining, citrate buffer 10 mM supplemented with 0.05% Tween 20 (pH 6.0) was used as an epitope retrieval buffer. The paraffin embedded sections were stained with Martius Scarlet Blue (MSB) technique, which includes three types of coloring agents; Martius yellow, crystal ponceau 6R, and aniline blue. Weigert's iron haematoxylin was used for nuclear staining. Four μm paraffin sections were stained with Picro Sirius Red to analyse cardiac fibrosis. All fluorescent images were taken on a confocal microscope (Zeiss LSM 700); all brightfield images were taken on an Axiovert 200 M light microscope (Zeiss).

### *In situ* hybridization

*In situ* hybridization was performed using SARS-CoV-2 probe synthesis primers (5'-ATT AAC CCT CAC TAA AGG GAT TTG GTG GAC CCT CAG ATT C-3' and 5'-TAA TAC GAC TCA CTA TAG GGG CGC GAC ATT CCG AAG AA-3'). These primers include polymerase binding sites for T3 polymerase in the forward primer (production of sense probe) and T7 polymerase in the reverse primer (production of anti-sense probe). For the staining, after slides were deparaffinized in xylene and rehydrated through a series of graded ethanol, tissue permeabilization was performed for 5 min in Proteinase K (3115828001, Sigma) at 37°C, followed by post-fixation in 4% PFA for 20 min. Slides were incubated with the antisense and sense probes overnight at 70°C, with a final concentration of 1 μg/ml. Slides were washed through a series of washing buffers and treated with 100 μg/mL RNAse A (EN0531, Thermofisher) for two times 15 min. Slides were blocked with 2% Blocking Reagent (11096176001, Roche) for 1 h and incubated overnight with anti-digoxigenin-AP Fab fragments antibody (1:2000, 11093274910, Roche) at 4°C. Slides were washed through a series of washing buffers and staining was visualized by BM purple AP substrate (11442074001, Roche) at room temperature, until staining was sufficient.

### Western blot

All Western blots were performed using standard procedures. Briefly, ~5–30 mg of tissue from the heart was lysed in 175 μL RIPA buffer. Lysate was centrifuged and the supernatant was run on a 10% SDS-PAGE gel and transferred to a nitrocellulose membrane. Membranes were blocked in skim milk for 1 h before being incubated with primary antibody (HIF1α, 1/500 in BSA) for 24 h at RT and HRP-conjugated secondary antibody (1/1,000). Blots were developed with ECL Prime Western Blotting Detection Reagent (Amersham) and imaged on a Bio-Rad ChemiDoc.

### Hypoxia treatment *in vitro*

Neonatal mouse ventricular cardiomyocytes were isolated on the day of birth (P0). Hearts were isolated and atria removed. Next, ventricles were mechanically minced before being put on multiple enzymatic digestion steps in digestion buffer [Pancreatine (Sigma), Collagenase type 2 (Gibco) and Dnase 1 (Invitrogen)]. After the final digestion step, cells were filtered through a 100 μM cell strainer and pre-plated for 1 h at 37°C, allowing non-cardiomyocytes to adhere. Finally, the medium containing the cardiomyocytes was collected and after centrifugation, cardiomyocytes were plated in fresh medium on gelatin-coated 24-well plates. Vero E6 cells (African green monkey kidney, ATCC CRL-1586) were cultured in minimal essential medium (15188319, Gibco) supplemented with 10% fetal bovine serum, 1% L-glutamine (25030149, Gibco), and 1% bicarbonate (25080094, Gibco). Cells were infected with SARS-CoV-2 strain BetaCov/Belgium/GHB-03021/2020 (EPI ISL 407976|2020-02-03)] at a multiplicity of 0.2 TCID_50_ per cell for 20 h and subsequently treated with 800μM Cobalt Chloride (CoCl_2_, C-8661, Sigma) for 6 h. Cells were fixed in 1% PFA overnight and stained with HIF1α (1/50) and Topro (1/1,000).

### Statistical analysis

Results are presented as mean ± SEM. Each data point represents the average value for one animal. Statistical analysis was performed using GraphPad 8 Prism. Variance was tested with F-test (*p* < 0.05) and normality was tested with Shapiro-Wilk test (*p* < 0.05). Biological outliers were picked up based on Grubb's test (*p* < 0.05). *In vivo* data were analyzed using a one-way ANOVA with Dunnett's *post hoc* test (*p* < 0.05 ^*^, 0.01 ^**^, 0.001 ^***^).

## Results

### SARS-CoV-2 infected hamsters present with diastolic dysfunction starting at 14 dpi

We studied the effect of SARS-CoV-2 infection on cardiac function, in an established COVID-19 hamster model (15). In this model, 12-week-old hamsters are inoculated intranasally with 2 × 10^6^ TCID_50_ SARS-CoV-2 virus, resulting in high viral replication in the lungs. This infection induces both local and systemic inflammation and is accompanied by clear histological and radiological signs of viral pneumonia, similar to COVID-19 patients (15). Viral replication in the lung peaks at 4 dpi, after which viral load decreases and the hamsters gradually recover. The model mimics the disease patterns observed in the majority of SARS-CoV-2 infected patients and is currently being used to investigate antiviral compounds and vaccines (15–18).

**Figure 1:**
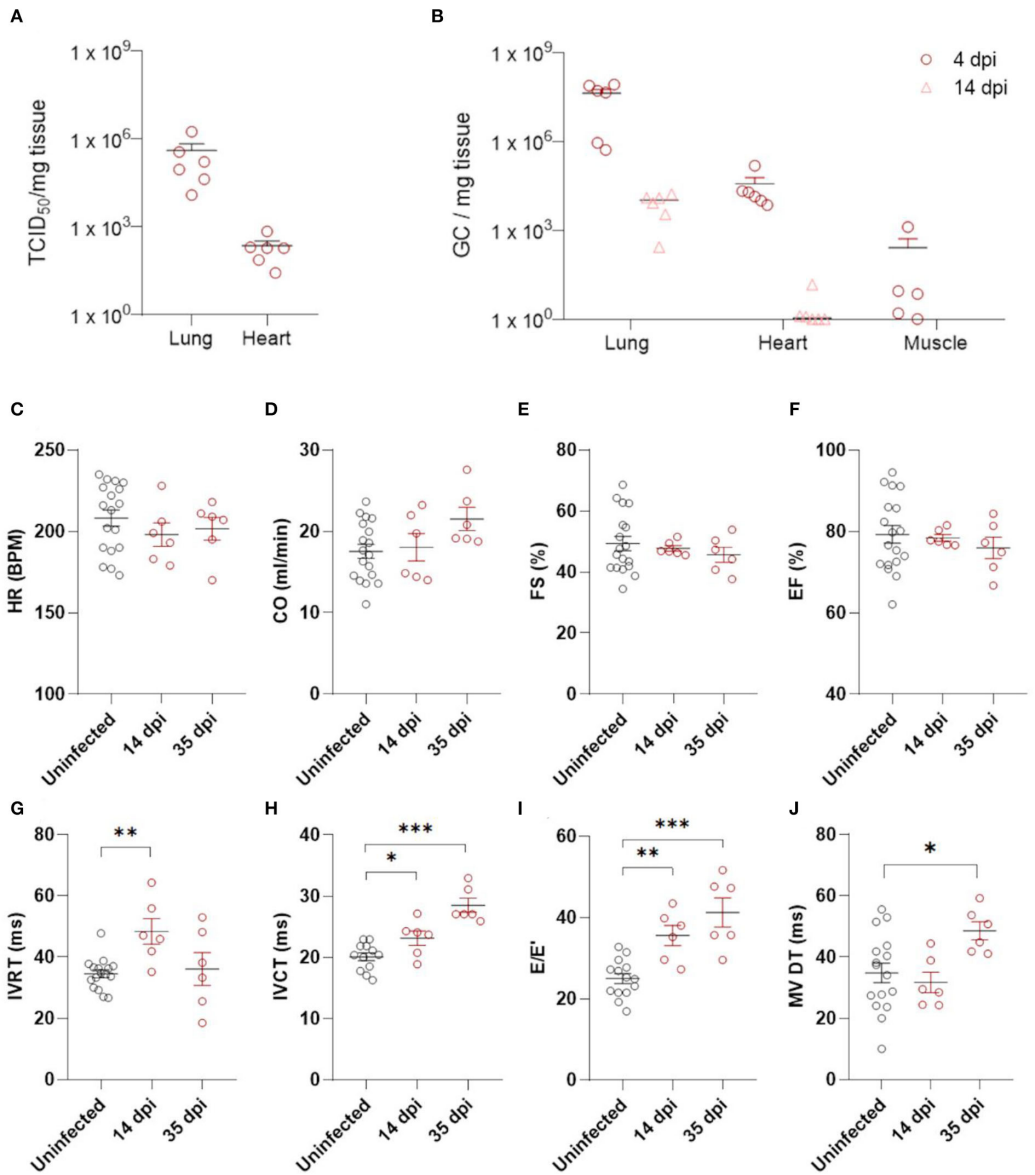
Echocardiography demonstrates diastolic dysfunction in SARS-CoV-2 infected hamsters. **(A)** Viral loads in tissue of hamsters 4 days post infection were assessed by endpoint dilution on cell cultures and expressed as TCID_50_ per mg of lung tissue. **(B)** Viral RNA levels were quantified by RT-qPCR in lung, heart, and muscle from infected and uninfected hamsters. **(C–J)** Cardiac function of 12 week old hamsters was investigated using echocardiography 2 and 5 weeks after SARS-CoV-2 infection, compared to age-matched controls. HR **(C)**, CO **(D)**, FS **(E)**, EF **(F)**, IVRT **(G)**, IVCT **(H)**, E/E′**(I)**, and MV DT **(J)** were analyzed. CO, cardiac output; DPI, days post Infection; E, early mitral inflow peak velocity; E′, early diastolic mitral annulus peak velocity; EF, ejection fraction; FS, fractional shortening; HR, heart rate; IVCT, isovolumic contraction time; IVRT, isovolumic relaxation time; MV DT, mitral valve deceleration time. Values are presented as mean ± SEM with *n* = 18 (uninfected) and *n* = 6 (SARS-CoV-2 infected hamsters). All data were analyzed using a one-way ANOVA followed by a Dunnett's multiple comparison test with * *p* < 0.05, ** *p* < 0.01, *** *p* < 0.001 comparing 14 dpi and 35 dpi hamsters to uninfected hamsters.

Using this model, we explored the consequences of SARS-CoV-2 infection on the heart. Titrations of homogenized cardiac tissue confirmed the presence of infectious particles in the heart at ~2.3 log10 TCID_50_/mg tissue, similar to reports from human biopsies (Figure 1A) (19). This compares to 4.1 log10 TCID_50_/mg tissue in the lungs (Figure 1A). Viral load, as assessed by RT-qPCR with endpoint titration, was highest in the lungs at 4 dpi and decreased, but was still present, by 14 dpi, compared to age-matched controls (Figure 1B). In the heart, infection had cleared by 14 dpi (Figure 1B). Muscle tissue at 4 dpi was used as control representing a tissue not significantly targeted by SARS-CoV-2 (Figure 1B). Similar to clinical data from SARS-CoV-2 patients, we observed no large areas of cardiomyocyte necrosis or immune cell infiltrations in the infected hamsters at any stage (Supplementary Figure 1) (20).

We assessed post-COVID-19 cardiac function by echocardiography at 14 and 35 dpi (Figures 1C–J, Supplementary Table 1). No differences in heart rate (HR) or cardiac output (CO) were observed (Figures 1C,D). Systolic function was assessed by measuring fractional shortening (FS) and ejection fraction (EF), but we observed no alterations (Figures 1E,F). A transient increase in isovolumetric relaxation time (IVRT) was present at 14 dpi in the infected hamsters compared to controls (Figure 1G). Interestingly, we observed an increase in E/E' and isovolumic contraction time (IVCT) at 14 dpi (Figures 1H,I), which remained at 35 dpi. At 35 dpi, the mitral valve deceleration time (MV DT) increased in infected hamsters compared to controls, further supporting diastolic dysfunction after SARS-CoV-2 infection (Figure 1J). E/A was unaffected by SARS-CoV-2 infection (Supplementary Table 1). However, A waves are difficult to measure accurately in rodents (21, 22), and often do not change in rodent models of diastolic dysfunction. Complete echocardiographic analysis is available in Supplementary Table 1. Taken together, our echocardiography data demonstrate that SARS-CoV-2 infected hamsters develop diastolic dysfunction weeks after recovering from COVID-19.

**Figure 2:**
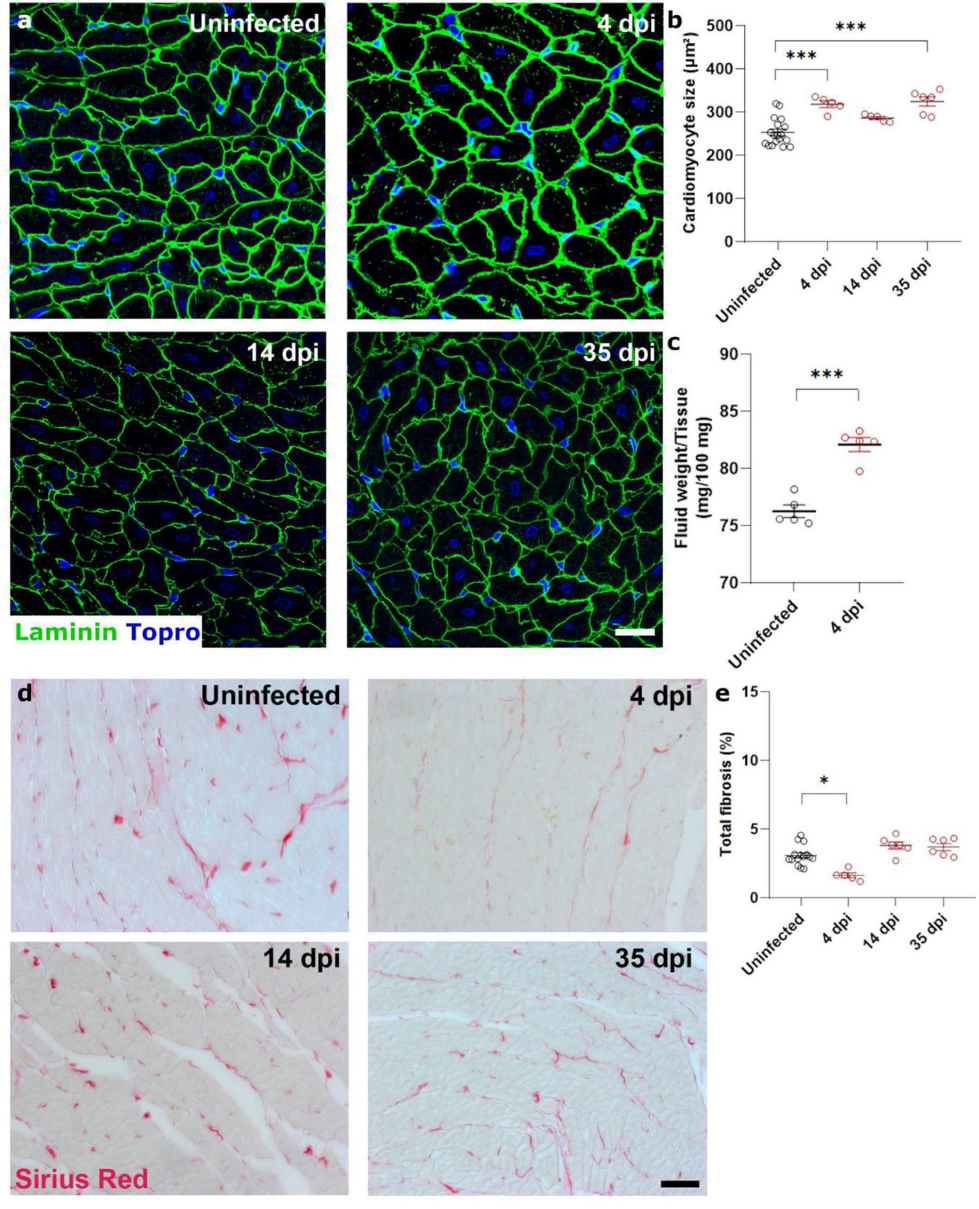
SARS-CoV-2 infected hamsters present with rapid and consistent cardiomyocyte swelling in the absence of interstitial fibrosis. Twelve week old hamsters were infected with SARS-CoV-2 virus and sacrificed 4 days post infection, at the peak of viral load, at 14 dpi, and at 35 dpi. **(a)** Left ventricle heart sections of age-matched control and infected hamsters were stained for laminin and Topro3 to quantify cardiomyocyte cell size **(b)**. **(c)** Cardiac wet weight and dry weight were compared to indicate cardiac oedema at 4 dpi. **(d)** Left ventricle heart sections of age-matched control and infected hamsters were stained with Picro Sirius Red to analyse cardiac fibrosis **(e)**. Values are presented as mean ± SEM with *n* = 18 (uninfected) and 5-6 (SARS-CoV-2 infected hamsters). All data were analyzed using a one-way ANOVA followed by a Dunnett's multiple comparison test with * *p* < 0.05, ** *p* < 0.01, *** *p* < 0.001 comparing 14 dpi and 35 dpi hamsters to uninfected hamsters. Scalebar represents 20 μm **(a)** and 50 μm **(d)**.

Diastolic heart failure describes the inability of the heart to properly relax during diastole, typically caused by a “stiffening” of the heart. In heart failure, this stiffening is a result from both increased matrix deposition and altered cardiomyocyte passive stiffness. As cardiomyocyte stiffness is associated with their size, we first assessed cardiomyocyte cross sectional area after SARS-CoV-2 infection. Surprisingly, cardiomyocytes already increased in size by 25% at 4 dpi, which did not resolve over time (Figures 2a,b). The timing was very rapid and unlikely to represent hypertrophic remodeling but was more akin to a cell swelling process. To confirm cardiac oedema, we compared wet weight vs. dry weight of the left ventricle. Indeed, at 4 dpi, SARS-CoV-2 infected hamster hearts held significantly more fluid per 100 mg tissue measured (82.1 ± 1.36 vs. 76.26 ± 1.10 mg per 100 mg tissue), confirming cardiac oedema (Figure 2c).

To investigate the presence of cardiac matrix deposition, we performed Sirius Red staining on sections of the hamster hearts (Figures 2d,e). We observed no differences in cardiac fibrosis in infected hamsters compared to uninfected hamsters. A small, but statistically significant, decrease was observed at 4 dpi, which can be attributed to the increased cardiomyocyte size which created an apparent reduction due to the reduced presence of intracellular space per unit area. However, we do not believe that this decrease has a biological relevance. Together, these data suggest that the observed diastolic dysfunction is likely secondary to general cardiac oedema or cell swelling.

### SARS-CoV-2 infection causes microvascular dysfunction, which results in signs of microvascular ischemia-reperfusion-like injury in the heart

**Figure 3:**
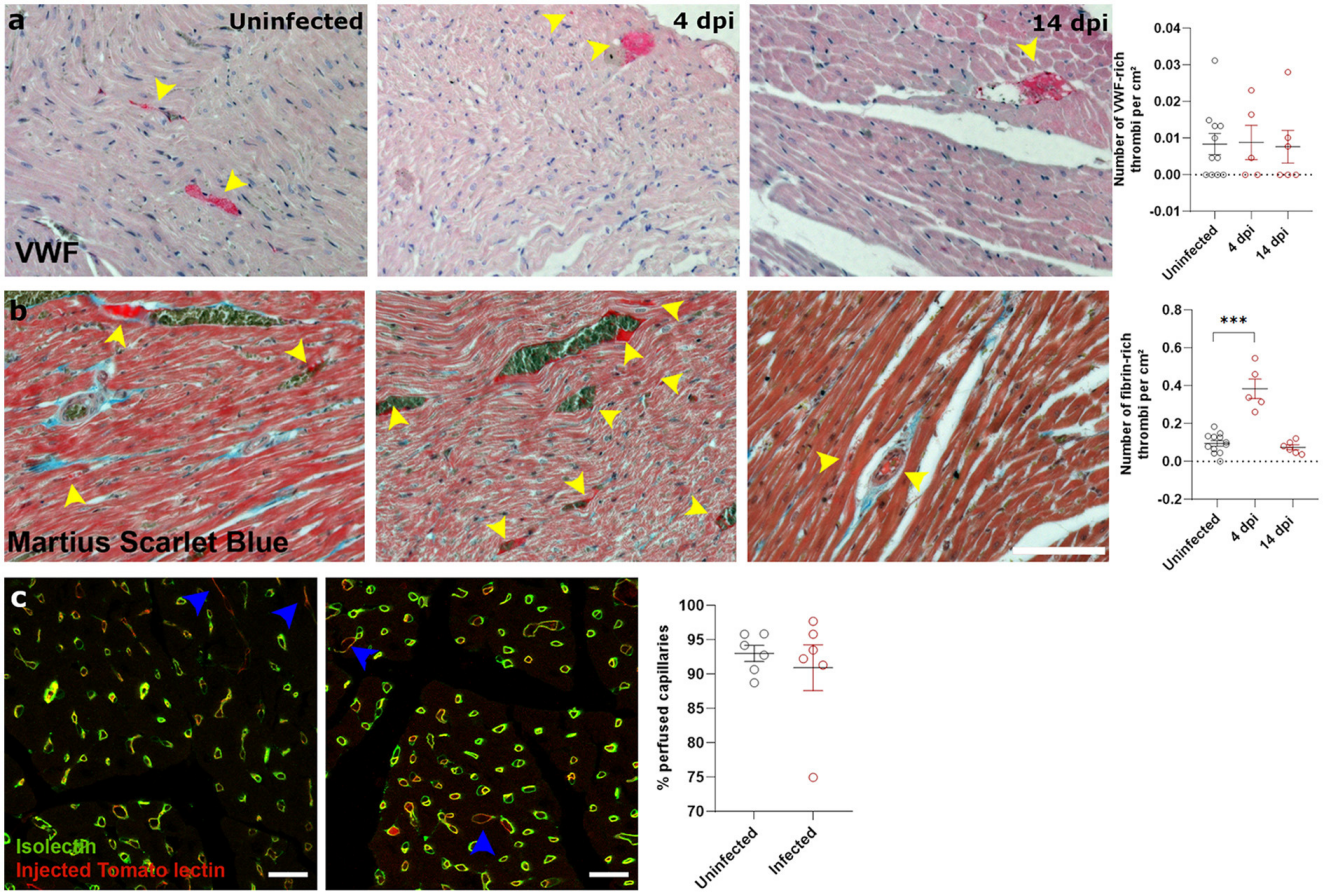
SARS-CoV-2 infected hamsters have transient fibrin-rich thrombi, while perfusion is not affected. **(a,b)** Twelve week old hamsters were infected with SARS-CoV-2 virus and sacrificed at 4 and 14 dpi. Left ventricle heart sections were stained for VWF **(a)** or Martius Scarlet blue **(b)** to analyse the number of VWF-rich and fibrin-rich thrombi, respectively. Yellow arrowheads indicate thrombi. Values are presented as mean ± SEM with *n* = 12 (uninfected) and 5-6 (SARS-CoV-2 infected hamsters). Statistical analysis was performed using a one-way ANOVA followed by a Dunnett's multiple comparison test with *** *p* < 0.001 comparing 4 dpi and 14 dpi hamsters to age-matched uninfected hamsters. **(c)** Hamsters were infected with SARS-CoV-2 virus and at 4 dpi injected with Tomato-Lectin 1 min before sacrifice to label perfused capillaries. Left ventricle heart sections were stained for isolectin B4 to analyse obstructed capillaries. Values are presented as mean SEM with *n* = 6. Statistical analysis was performed using a two-tailed Student's *t*-test.

Next, we investigated how SARS-CoV-2 infection induced the observed cardiac oedema. Cardiomyocyte swelling occurs in the initial stage of ischemia-reperfusion (23). We therefore investigated whether the heart was ischemic in SARS-CoV-2 infected hamsters. Since microthrombi have often been reported in COVID-19 patients (20, 24), we investigated the presence of cardiac microthrombi to explain the observed increase in cardiomyocyte size. We observed no difference in the number of von Willebrand Factor (vWF)-rich thrombi in infected hearts compared to the control hearts, at any time point (Figure 3a). A transient increase in fibrin-rich thrombi, as assessed with Martius Scarlet Blue staining, was present at 4 dpi and resolved by 14 dpi (Figure 3b). The presence of microthrombi did not cause a complete microvascular occlusion, as injection of Tomato-lectin to label perfused vessels shortly before sacrifice indicated no difference between groups (Figure 3c). However, tomato-lectin injection can only detect complete occlusion of a vessel and is insensitive to more subtle levels of hypoperfusion, which could go undetected. Similarly, fibrin-rich non-occlusive cardiac thrombi has also been observed extensively in patients (20, 24).

**Figure 4:**
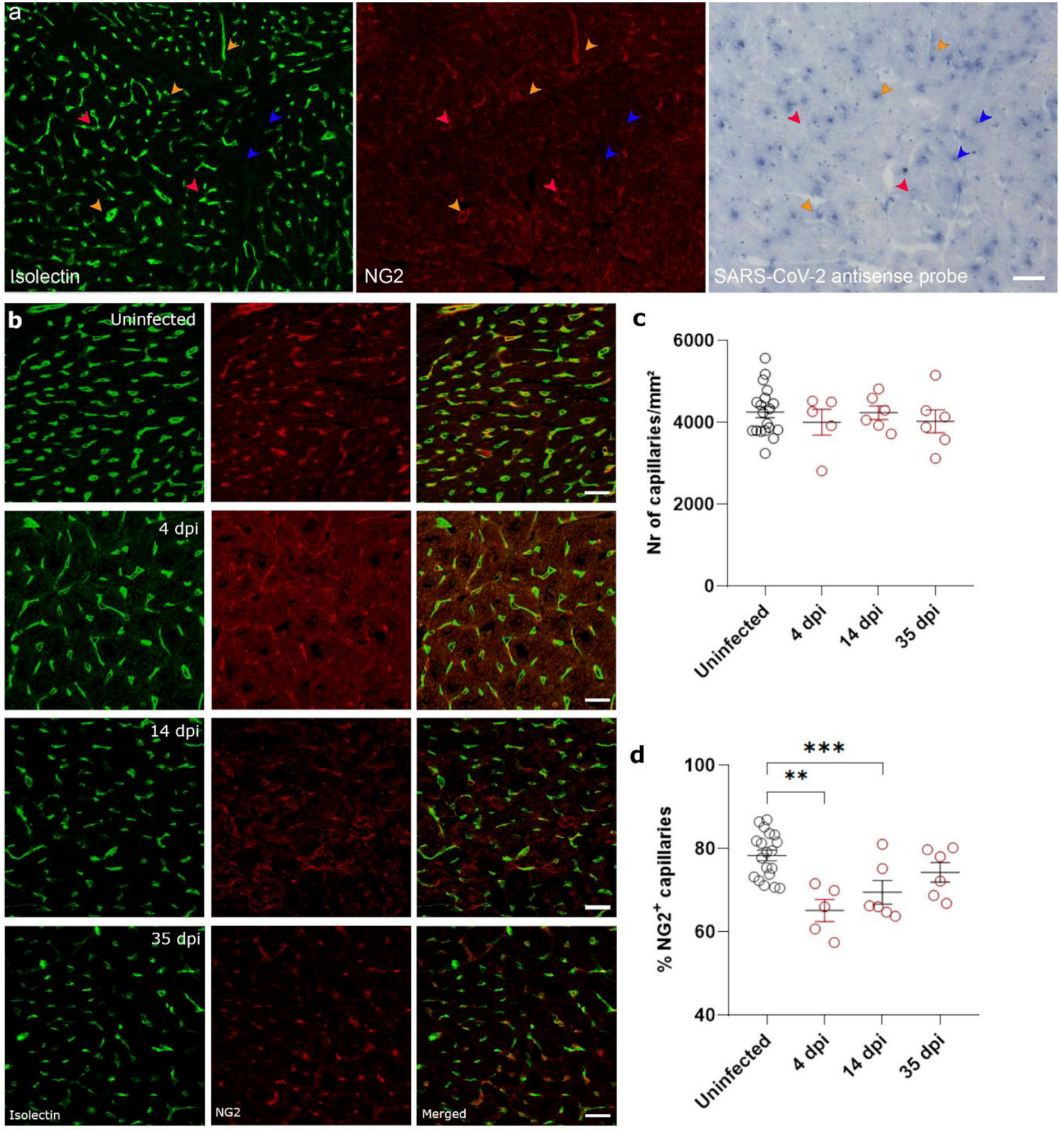
SARS-CoV-2 infected hamsters lose their NG2^+^ pericytes at 4 dpi. Twelve week old hamsters were infected with SARS-CoV-2 virus and sacrificed 4 dpi, at the peak of viral load, 14 dpi, and 35 dpi. **(a)** SARS-CoV-2 infection was analyzed by *in situ* hybridization techniques in the left ventricle. Infected pericytes (yellow arrowhead), uninfected pericytes (red arrowhead), and other cell types that are infected (blue arrowheads) were present. **(b–d)** Left ventricle heart sections of age-matched control and infected hamsters were stained for Isolectin (green) and NG2 (red) to analyse the percentage of capillaries covered in pericytes **(d)**. NG2, neuron-glial antigen 2. Values are presented as mean ± SEM with *n* = 18 (uninfected) and 5-6 (SARS-CoV-2 infected hamsters). All data were analyzed using a one-way ANOVA followed by a Dunnett's multiple comparison test with *** *p* < 0.001 comparing 14 dpi and 35 dpi hamsters to age-matched uninfected hamsters. Scalebar represents 50 μm **(a)** and 20 μm **(b)**.

Ischemia-reperfusion injury is often associated with microvascular dysfunction, such as increased permeability of capillaries and hypoperfusion. Both the control of microvascular flow and vessel permeability are heavily influenced by pericytes (25). Extensive loss of capillary pericytes have been reported in the lungs of severe COVID-19 patients (26). In heart (27, 28) and brain tissue (28, 29), pericytes express ACE2, rendering them a likely target of cardiac SARS-CoV-2 infection. *In situ* hybridization techniques on 4 dpi hearts and stained for endothelial cells (isolectin B4) and pericytes (NG2 and colocalization with capillaries) on serial sections showed that some, but not all, pericytes were infected with SARS-CoV-2 (Figure 4a, Supplementary Figure 2). Although viral particles have been observed by TEM in endothelial cells (20) and cardiomyocytes (19), *in situ* hybridization techniques are more sensitive to an actively replicating virus. Others have also reported that the virus does not replicate in endothelial cells (30).

Given the presence of virus in cardiac pericytes, we investigated cardiac microvascular changes in infected hamster hearts at 4 dpi. Infected hearts present with a significant loss in NG2^+^ pericyte coverage of capillaries at 4 dpi and 14 dpi (Figures 4b,d). By 35 dpi, the capillary coverage of NG2^+^ pericytes was almost completely recovered. These changes in cardiac pericyte coverage were not associated with any changes in microvascular density at any of the timepoints (Figure 4c). Thus, these data suggest that both the presence of microthrombi and loss of pericytes results in microvascular dysfunction, which in turn will lead to cardiomyocyte swelling.

### SARS-CoV-2 infection causes hypoxia in cardiomyocytes and prevents the translocation of HIF1α to the nucleus

**Figure 5:**
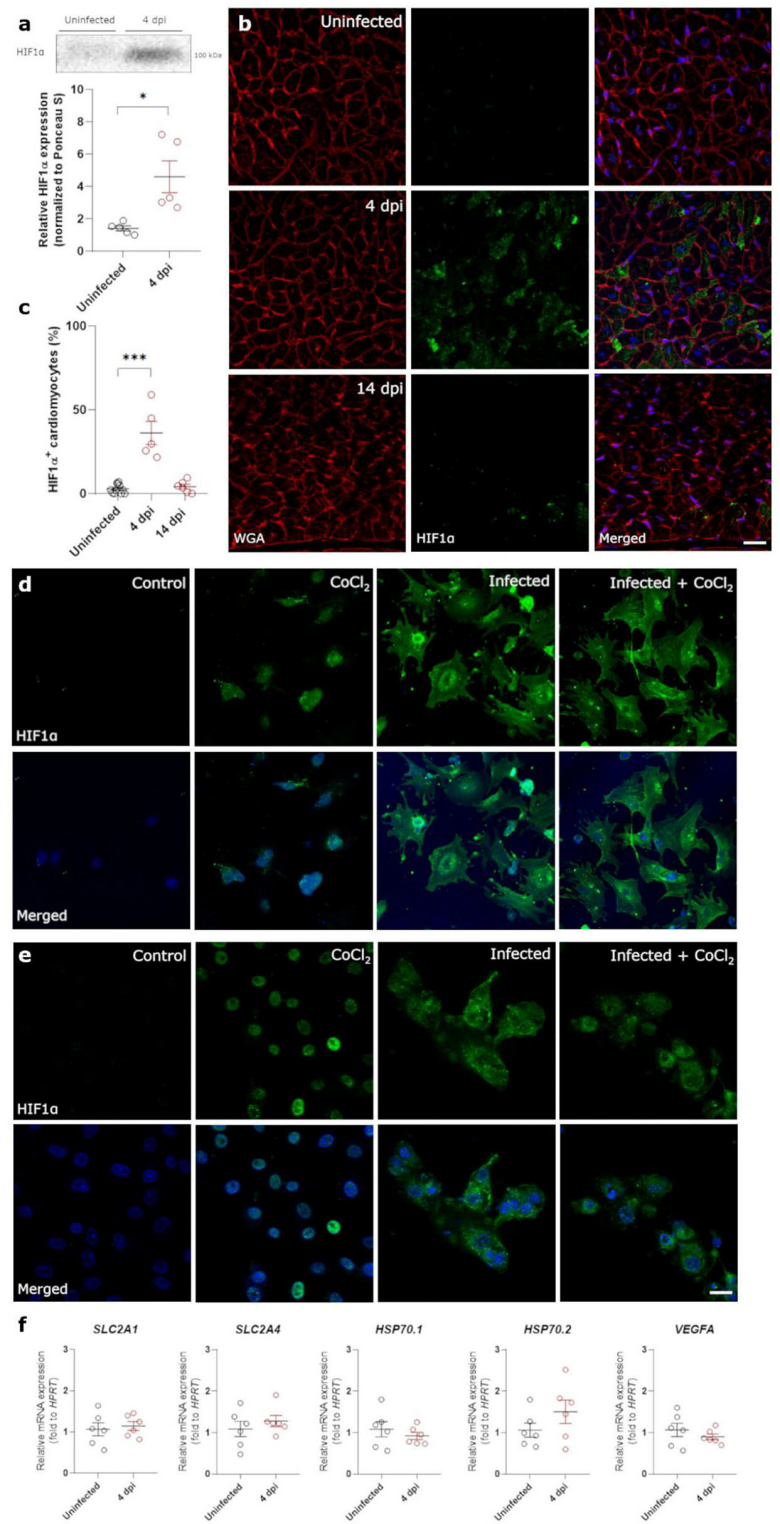
SARS-CoV-2 infection prevents translocation of HIF1α in hamster hearts and *in vitro*. Twelve week old hamsters were infected with SARS-CoV-2 virus and sacrificed at 4 dpi, at the peak of viral load, and at 14 dpi. **(a)** HIF1α expression in protein lysates of 4 dpi hamster hearts was analyzed using western blot. Values are normalized to Ponceau S staining of total protein with *n* = 6. Statistical analysis was performed using a two-tailed Student's *t*-test with * *p* < 0.05. **(b)** Left ventricle heart sections of age-matched control and infected hamsters were stained for HIF1α, WGA, and Topro3 to quantify HIF1α positive cardiomyocytes **(c)**. Values are presented as mean ± SEM with *n* = 18 (uninfected) and 5-6 (SARS-CoV-2 infected hamsters). Data were analyzed using a one-way ANOVA followed by a Dunnett's multiple comparison test with * *p* < 0.05, *** *p* < 0.001 comparing 4 dpi and 14 dpi hamsters to age-matched uninfected hamsters. **(d,e)** Freshly isolated cardiomyocytes **(d)** and Vero E6 cells **(e)** were infected with the SARS-CoV-2 virus at a concentration of 0.2 TCID_50_ per cell for 20h and subsequently treated with 800 μM CoCl_2_ for 6h *in vitro*. Cells were stained for HIF1α and Topro3 to analyse the translocation of HIF1α in a hypoxic environment. **(f)** Total RNA was isolated from infected hamster hearts at 4 dpi and RNA levels for *SLC2A1, SLC2A4, HSP70*.1, *HSP70*.2, and *VEGFA* were analyzed by RT-qPCR. Values are presented as mean ± SEM with *n* = 5–6. Statistical analysis was performed using a two-tailed students *t*-test. CoCl_2_, Cobalt Chloride; HIF1α, Hypoxia Inducible Factor 1α; HSP70, Heat Shock protein 70; SLC2A1, Solute Carrier Family 2 Member 1; SLC2A4, Solute Carrier Family 2 Member 4; VEGFA, vascular endothelial growth factor A; WGA, Wheat Germ Agglutinin. Scalebar represents 20 μm **(b)** and 10 μm **(d)**.

Both non-occlusive fibrin-rich microthrombi and the loss of pericyte coverage in the heart cause reduced perfusion and hypoxia. Therefore, we investigated the presence of Hypoxia Inducible Factor 1α (HIF1α) within the left ventricle of control and infected hamster hearts. Western blot analysis of lysates of heart samples showed that HIF1α expression was highly upregulated at 4 dpi (Figure 5a, Supplementary Figure 3). HIF1α stabilization has previously been reported after infection with other viruses (16, 31). However, HIF1α was not present within the nuclei as expected, but was strongly expressed within the cytoplasm of cardiomyocytes at 4 dpi compared to control (Figures 5b,c). At 14 dpi, HIF1α staining was absent, coinciding with the end of cardiac infection, pericyte recovery, and the fibrin-rich thrombi being resolved (Figures 5b,c). SARS-CoV-1 virus has previously been shown to inhibit STAT1 translocation (32), and we hypothesized that the cytoplasmic pattern may be due to an inhibition of translocation after infection. To test this hypothesis, we infected freshly isolated cardiomyocytes and Vero E6 cells *in vitro* in the presence of CoCl_2_, a known inducer of hypoxia and HIF1α (Figures 5d,e). Vero cells were used to assess whether the effect was specific to cardiomyocytes, or also occurred in other cell types. As expected, cells treated with CoCl_2_ express HIF1α strongly within the nucleus. SARS-CoV-2 infected cells, both in the presence and absence of CoCl_2_, show high levels of HIF1α expression, however, it fails to translocate to the nucleus and remains within the cytoplasm in both cell types. Given that HIF1α was not reaching the nucleus, we further confirmed that HIF1α target genes were not expressed (Figure 5f). None of the HIF1α target genes were upregulated at 4 dpi, demonstrating that the HIF1α-regulated pathway is disrupted in response to SARS-CoV-2 infection. These data suggest that SARS-CoV-2 infection prevents the translocation of HIF1α to the nucleus in response to hypoxia.

## Discussion

Recovered COVID-19 patients show signs of cardiac injury (12) and in some cases develop viral myocarditis-like syndromes (33). In our hamster model, SARS-CoV-2 infection induces an increase in E/E', IVCT, and MV DT, which are measures of diastolic dysfunction in rodents. In the clinic, 13.2% of hospitalized patients present with diastolic dysfunction, without prior history (5). The underlying mechanism by which SARS-CoV-2 infection causes cardiac injury remained poorly understood. Diastolic dysfunction is a result of a stiffening of the heart and the loss of cardiomyocyte contractility. In our SARS-CoV-2 infected hamsters, cardiomyocytes increased in size almost immediately after infection. This rapid increase persisted over 5 weeks, after hamster had recovered from SARS-CoV-2 infection, while we observed no signs of cardiac fibrosis. Moreover, wet weight vs. dry weight comparison demonstrated that SARS-CoV-2 infected hamsters suffered cardiac oedema at 4 dpi. Therefore, we suggest that SARS-CoV-2 infection induces cell swelling rather than hypertrophic remodeling. Our results support the previous notion that the cardiac phenotype of COVID-19 in patients represents a “swollen heart” (34).

The observed cardiomyocyte swelling and cardiac oedema are reminiscent of microvascular ischemia-reperfusion injury. We found that cardiomyocytes remain enlarged even when the virus is no longer present (14 dpi, Figure 1B). Therefore, cardiomyocyte swelling cannot be a direct effect of SARS-CoV-2 infection, but must be secondary or reinforced by other aspects of the pathology. We observed the presence of transient, non-occlusive fibrin-rich microthrombi, suggesting hypoperfusion occurs in response to SARS-CoV-2 infection (Figure 3). Furthermore, non-occlusive cardiac thrombi can increase resistance to flow in certain vessels. At the same time, pericyte coverage is reduced in the microvasculature (Figure 4). Moreover, severe pericyte loss is observed in lung tissue of COVID-19 patients, causing the observed micro-vasculopathy (35). Pericytes control both microvascular flow and vessel permeability (25). As pericytes provide a baseline tone for capillaries, a loss of pericytes induces capillary dilatation (36). Although local loss of pericyte tone increases blood flow to a certain region (37), massive loss of microvascular tone induces reductions in blood flow (38). In patients, COVID-19 has been associated with microvascular dilatation (39, 40). Thus, we suggest that the loss of pericytes as a result of SARS-CoV-2 infection causes microvascular dysfunction (41).

In our hamster model, we observed the SARS-CoV-2 virus infecting cardiac pericytes and subsequent reduction of cardiac pericyte coverage. However, whether the SARS-CoV-2 is able to infect vascular cells is still up for debate. Recent findings suggest that cardiac pericytes are not homogeneously infected *in vitro*, but rather the spike protein affects pericytes through binding to the CD147 receptor (42). In turn, the spike protein induces pericyte motility and impairs pericyte-endothelial cross talk (42). Such an impaired pericyte-endothelial cross talk could result in a detachment of pericytes from the microvasculature and the observed reduced pericyte coverage. However, we did not elucidate those specific mechanisms in this study.

Given that pericytes recover, microthrombi resolve and HIF1α can translocate once the virus is cleared, we expect that cardiac swelling would resolve over time. However, patients with a reduced pericyte coverage before SARS-CoV-2 infection, such as dementia patients (43), or reduced capacity for pericyte regeneration may not be able to recover as easily. Patients with dementia show increased mortality rates after SARS-CoV-2 infection (44–46), which might be linked to reduced pericyte coverage before SARS-CoV-2 infection that renders them more prone to the effect of infection on their remaining pericytes.

In severe cases of COVID-19, hypoxia has been reported abundantly and is suggested to be the main cause of mortality (47). Moreover, HIF1α signaling is dysregulated, as its mRNA levels are upregulated in blood samples from patients suffering from COVID-19 (48). In these patients, HIF1α activation has been proposed to initiate a cytokine storm, which can cause severe implications, such as pneumonia and ARDS, that eventually result in organ failure, or even death (31). In COVID-19 patients, we suspect that the presence of SARS-CoV-2 virus in the heart has a similar effect on HIF1α stabilization. In general, loss of HIF1α signaling is associated with an infection response that is low in neutrophils and high in eosinophils (49). The opposite is seen in hospitalized COVID-19 patients (50–52). Thus, we propose that this inhibition of HIF1α translocation occurs early and results in a failure to adapt to the hypoxia, which is accompanied by the ongoing HIF1α accumulation. Our data clearly demonstrate that once the SARS-CoV-2 virus is no longer present, normal HIF1α signaling proceeds. This clearance happens early on for most organs. We propose that when HIF1α can translocate again after virus clearance, the early upregulation of HIF1α results in an exaggerated hypoxia response. Though ideally, we would validate this in patient samples, this would require cardiac biopsies on patients in the early stage of disease, before they are even hospitalized.

Though HIF1α is stabilized with SARS-CoV-2 infection, our results show that it does not activate its downstream target. SARS-CoV-2 also prevents translocation of the transcription factor to the nucleus. This translocation is necessary to efficiently resolve hypoxia and ensure cell survival. Therefore, we suggest that preventing HIF1α translocation ensures its prolonged presence and maintains SARS-CoV-2 replication. Moreover, our data support the notion that inhibiting HIF1α translocation is a general consequence of SARS-CoV-2 infection, rather than a cardiomyocyte-specific phenotype (Figure 5). In severe COVID-19 cases, preventing HIF1α degradation could worsen the immune response, of which the implications are unknown and could be severe. Thus, our data reveal a potential strategy of the SARS-CoV-2 virus to activate the innate immune response.

Even among patients that are not hospitalized, symptoms often persist even months after their initial recovery (53). In our hamster model, diastolic dysfunction developed long after hamsters recovered from SARS-CoV-2 infection. In long-covid patients, a subgroup of patients suffers from cardiac related syndromes, such as Postural Tachycardia Syndrome (PoTS), a chronic condition characterized by a consistent orthostatic tachycardia upon standing. Patients develop PoTS months after their SARS-CoV-2 infection, without a prior history. Recently, a link with endothelial dysfunction has been made (54). PoTS patients are reported to have reduced flow-mediated dilations compared to healthy controls (54). In our hamster model, SARS-CoV-2 infection reduces pericyte coverage and impairs their function *in vitro*. We suggest that SARS-CoV-2 infection induces microvascular dysfunction, which might initiate the onset of long-covid syndromes, such as PoTS. Moreover, a preliminary study suggests that SARS-CoV-2 infection induces microvascular dysfunction in long-covid patients that suffered mild to moderate COVID-19. Half of these patients showed a significant circumferential subendocardial perfusion defect, which suggests microvascular dysfunction, based on a CMR adenosine stress test. Thus, we suggest SARS-CoV-2 infection initiates microvascular dysfunction, which may have severe effects on the long-term.

Overall, our results show that SARS-CoV-2 infection is most likely to affect vascular flow patterns, causing an microvascular ischemia-perfusion-like event. We propose that this is caused by regional variations in blood flow, resulting from partial occlusion by microthrombi and microvascular dilation caused by microvascular dysfunction. As hypoxia is not resolved efficiently, these events result in a stiffer/oedemic heart and diastolic dysfunction. As such, COVID-19 may be an independent risk factor for the development of diastolic dysfunction (55).

## Data availability statement

The raw data supporting the conclusions of this article will be made available by the authors, without undue reservation.

## Ethics statement

The animal study was reviewed and approved by Animal Care and Use Committee of KU Leuven.

## Author contributions

MD performed experiments, designed experiments, analysed data, and wrote the manuscript. LL performed experiments, designed experiments, analysed data, and edited the manuscript. IC and JR analysed data and edited the manuscript. RB and SJS performed experiments and edited the manuscript. SK performed experiments, analysed data, and edited the manuscript. JVW, NG, TVB, and ML performed experiments. PV and SVL designed experiments and edited the manuscript. KM and SH designed experiments and edited the manuscript. CT performed experiments, designed experiments, and edited the manuscript. EAVJ designed experiments, analysed data, and wrote the manuscript. All authors contributed to the article and approved the submitted version.

## Funding

This work was supported by the Fonds Wetenschappelijk Onderzoek [G091018N and G0B5920N to EJ, 1160718N to IC, 1107721N to MD, and G0G4820N to JN], the European Union's Horizon 2020 research and innovation program [848109 to EJ and 101003627 to JN], the COVID-19-Fund KU Leuven/University Hospitals Leuven to JN, and the Bill and Melinda Gates Foundation [INV-00636 to JN].

## Conflict of interest

The authors declare that the research was conducted in the absence of any commercial or financial relationships that could be construed as a potential conflict of interest.

## Publisher's note

All claims expressed in this article are solely those of the authors and do not necessarily represent those of their affiliated organizations, or those of the publisher, the editors and the reviewers. Any product that may be evaluated in this article, or claim that may be made by its manufacturer, is not guaranteed or endorsed by the publisher.
