# Supplementary material for: SARS-CoV-2 infection causes prolonged cardiomyocyte swelling and inhibition of HIF1α translocation in an animal model COVID-19
Source: Front Cardiovasc Med. 2022 Oct 17;9:964512. doi: 10.3389/fcvm.2022.964512 (PMC9618878; doi:10.3389/fcvm.2022.964512)
Supplement: Supplementary file 1 [file Data_Sheet_1.pdf]

SUPPLEMENTAL FIGURES

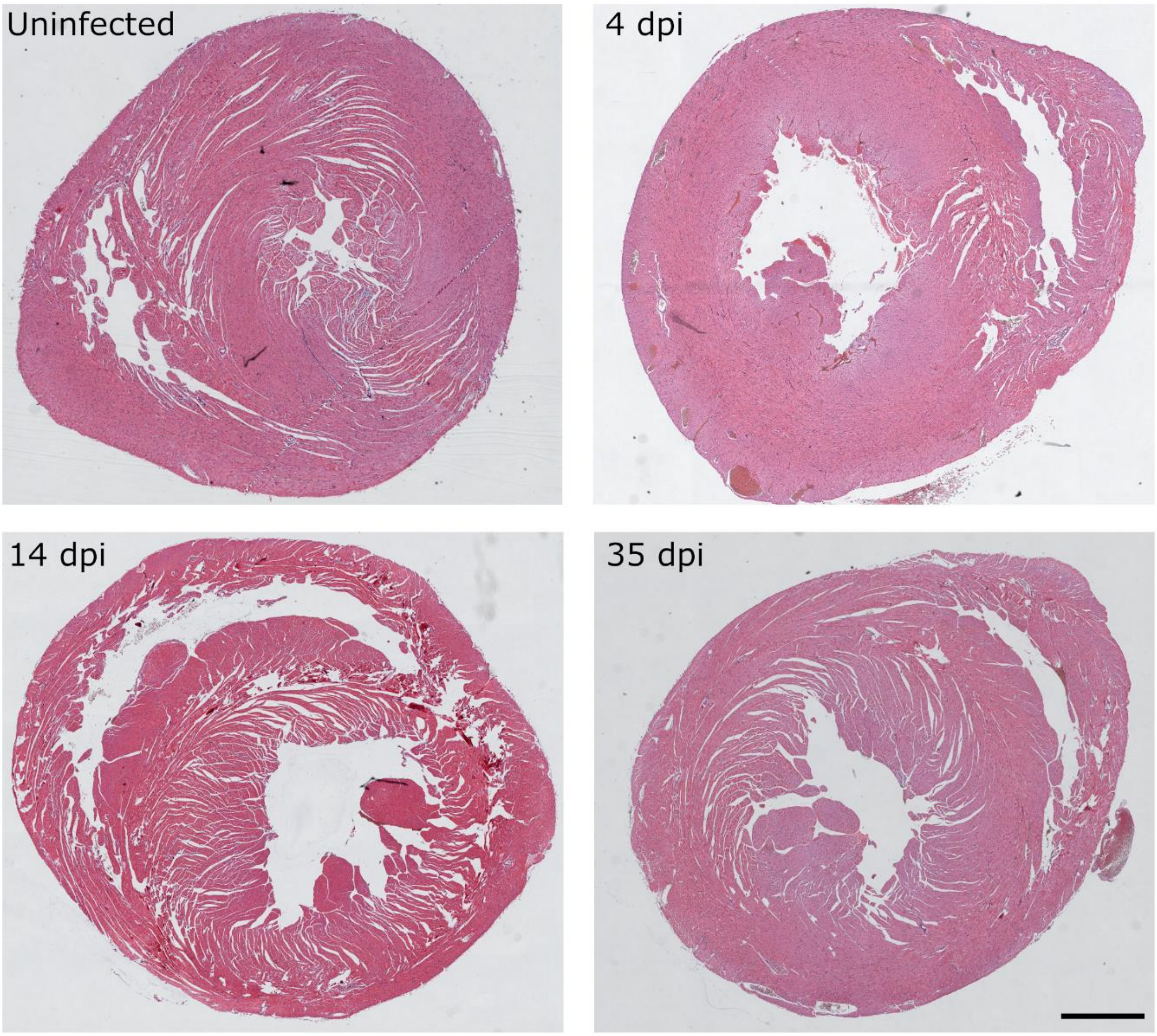

Supplemental Figure 1. SARS-CoV-2 infected hamsters show no signs of immune cell infiltration or cardiac necrosis. 8-week-old hamsters were infected with SARS-CoV-2 virus and sacrificed at 4, 14, and 35 days after infection. Heart sections were stained for haematoxylin and eosin. Scalebar represents 1 mm.

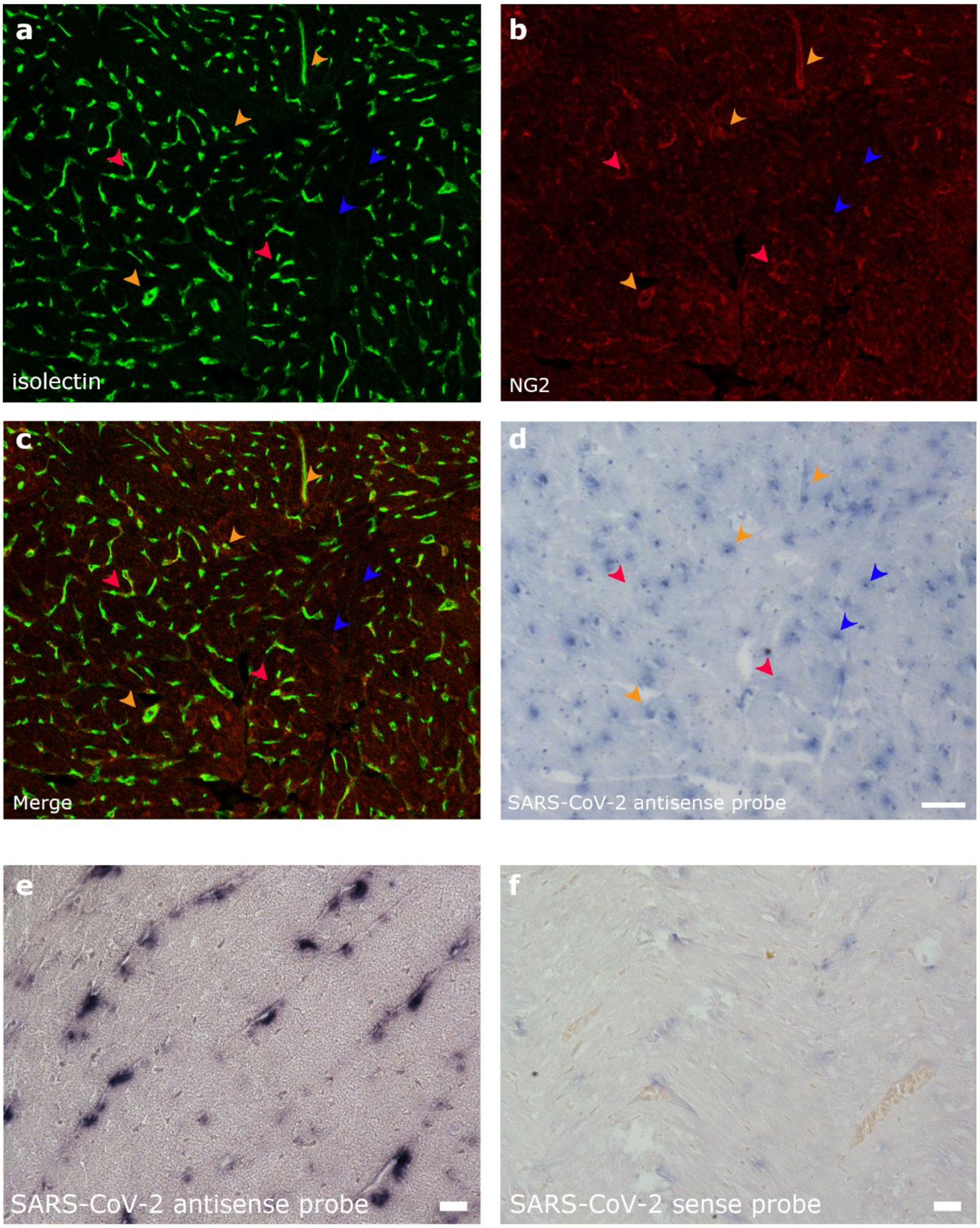
a
isolectin
b
NG2
c
Merge
d
SARS-CoV-2 antisense probe
e
SARS-CoV-2 antisense probe
f
SARS-CoV-2 sense probe

Supplemental Figure 2. Hamsters were infected with SARS-CoV-2 virus and sacrificed 4 days post infection, at the peak of viral load. (**a-d**) SARS-CoV-2 infection was analysed by *in situ* hybridization techniques. Heart sections of control and infected hamsters were stained for isolectin (**a**) and NG2 (**b**) and viral RNA levels (**d**) to analyse the SARS-CoV-2 infection of pericytes. Infected pericytes (yellow arrowhead), uninfected pericytes (red arrowhead), and other cell types that are infected (blue arrowheads) were present. (**e-f**) Heart sections were analysed by *in situ* hybridization anti-sense and control sense probes (**f**) NG2, neuron-glial antigen 2. Scalebar represents 50 µm (**a-f**).

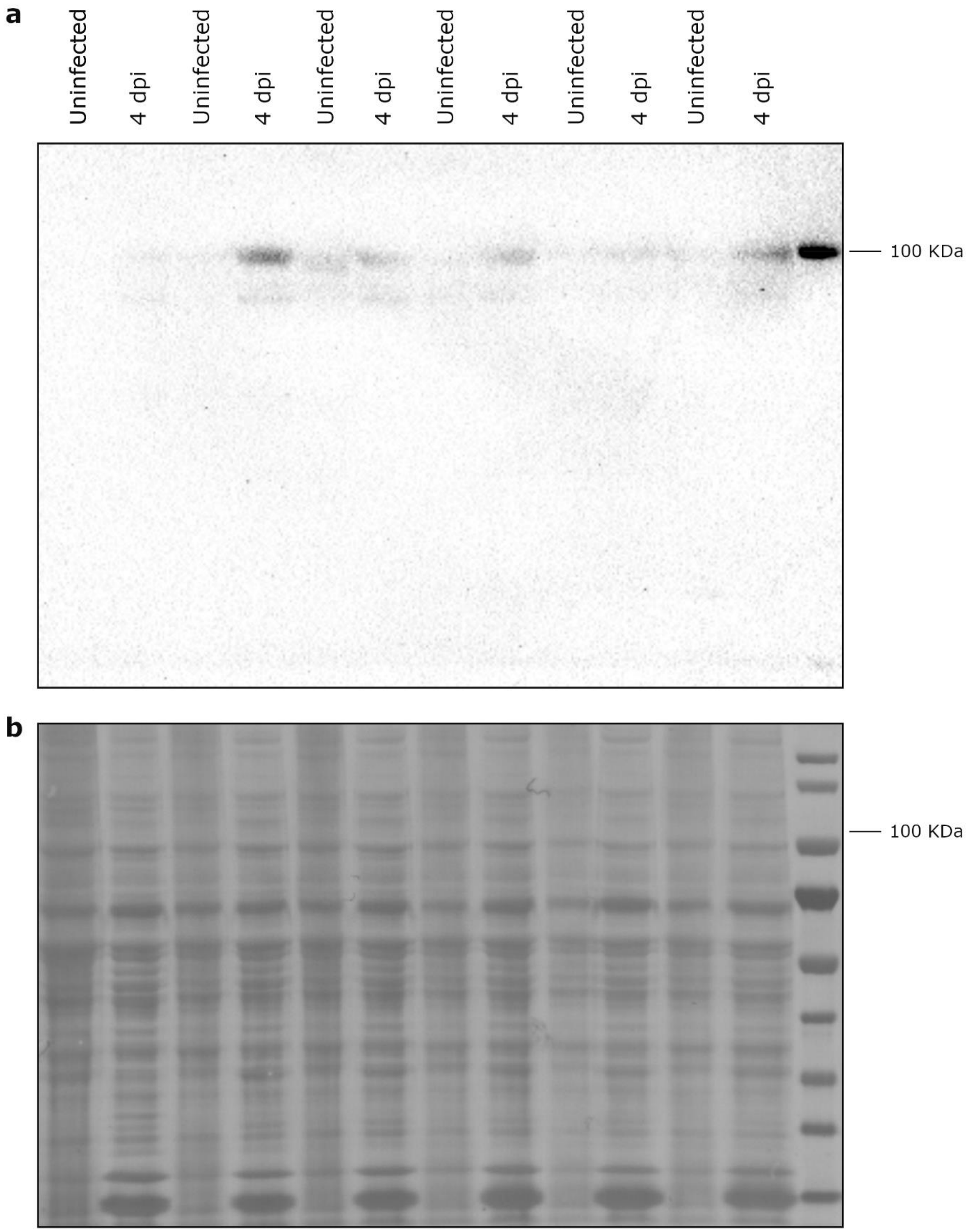

Supplemental Figure 3. (a) Full western blot membrane with HIF1α present at 100 kDa. (**b**) Corresponding Ponceau S image used for normalization and loading control.
