# Supplementary material for: SARS-CoV-2 infection causes prolonged cardiomyocyte swelling and inhibition of HIF1α translocation in an animal model COVID-19
Source: Front Cardiovasc Med. 2022 Oct 17;9:964512. doi: 10.3389/fcvm.2022.964512 (PMC9618878; doi:10.3389/fcvm.2022.964512)
Supplement: Supplementary file 2 [file Table_1.pdf]

**Supplemental Table 1. Echocardiography parameters in uninfected and 14 dpi and 35 dpi hamsters.**

| Parameter | Uninfected (n=18) | Infected 14 dpi (n=6) | Infected 35 dpi (n=6) |
|---|---|---|---|
| **HR (bpm)** | 208 ± 4.9 | 198 ± 7.2 | 202 ± 7.0 |
| **SV (µl)** | 93 ± 5.2 | 92 ± 8.8 | 107 ± 6.9$^{*}$ |
| **CO (ml/min)** | 17.6 ± 0.85 | 18 ± 1.7 | 21.5 ± 1.4 |
| **LV mass (mg)** | 484 ± 38 | 472 ± 52 | 396 ± 21 |
| **LV mass corrected (mg)** | 287 ± 25 | 378 ± 41 | 316 ± 16 |
| **$AW_d$ (mm)** | 1.8 ± 0.09 | 1.5 ± 0.13 | 1.57 ± 0.07 |
| **$AW_s$ (mm)** | 2.6 ± 0.08 | 2.5 ± 0.06 | 2.3 ± 0.07 |
| **EDD (mm)** | 4.8 ± 0.15 | 4.9 ± 0.22 | 5.4 ± 0.20 |
| **ESD (mm)** | 2.5 ± 0.17 | 2.6 ± 0.14 | 2.9 ± 0.22 |
| **$PW_d$ (mm)** | 1.7 ± 0.14 | 1.8 ± 0.22 | 1.1 ± 0.07 |
| **$PW_s$ (mm)** | 2.4 ± 0.14 | 2.3 ± 0.21 | 1.9 ± 0.07 |
| **EDV (µl)** | 110 ± 10 | 117 ± 12 | 142 ± 13 |
| **ESV (µl)** | 25 ± 4.2 | 25 ± 2.9 | 35 ± 6.8 |
| **EF (%)** | 79 ± 2.2 | 78 ± 0.83 | 76 ± 2.6 |
| **FS (%)** | 49 ± 2.3 | 48 ± 0.93 | 46 ± 2.5 |
| **E (mm/s)** | 792 ± 38 | 937 ± 63 | 919 ± 64 |
| **A (mm/s)** | 222 ± 14 | 207 ± 31 | 211 ± 20 |
| **IVRT (ms)** | 35 ± 1.3 | 48 ± 4.2$^{**}$ | 36 ± 5.4 |
| **MV DT (ms)** | 35 ± 3.2 | 32 ± 3.3 | 49 ± 2.9$^{*}$ |
| **IVCT (ms)** | 20 ± 0.54 | 23 ± 1.2$^{*}$ | 23 ± 1.2$^{***}$ |
| **AET (ms)** | 80 ± 2.5 | 108 ± 13$^{**}$ | 98 ± 4.3$^{**}$ |
| **NFT (ms)** | 136 ± 2.9 | 179 ± 17$^{***}$ | 163 ± 6.2$^{***}$ |
| **VTI (mm)** | 48 ± 2.9 | 51 ± 2.7 | 51 ± 1.9 |
| **MPI (AU)** | 0.72 ± 0.03 | 0.69 ± 0.05 | 0.67 ± 0.06 |
| **E’ (mm/s)** | 30 ± 1.4 | 27 ± 1.35 | 23 ± 1.9$^{*}$ |
| **A’ (mm/ms)** | 16 ± 1.0 | 20 ± 3.66 | 14 ± 2.4 |
| **S’ (mm/ms)** | 29 ± 1.5 | 33 ± 1.87 | 26 ± 2.1 |
| **E/A (AU)** | 3.7 ± 0.26 | 5.1 ± 0.80 | 4.49 ± 0.43 |
| **E/E’ (AU)** | 27 ± 1.8 | 36 ± 2.5$^{**}$ | 41 ± 3.6$^{***}$ |
| **E’/A’ (AU)** | 2.0 ± 0.13 | 1.5 ± 0.20 | 1.8 ± 0.36 |

Values are presented as mean ± SEM. A, late mitral inflow peak velocity; A’, late mitral annulus peak velocity; AET, aortic ejection time; AW, anterior wall; CO, cardiac output; d, in diastole; E, early mitral inflow peak velocity; E’, early diastolic mitral annulus peak velocity; EDD, end-diastolic diameter; EDV, end-diastolic volume; EF, ejection fraction; ESD, end-systolic diameter; ESV, end-systolic volume; FS, fractional shortening; HR, heart rate; IVCT, isovolumic contraction time; IVRT, isovolumic relaxation time; LV, left ventricular; MPI, myocardial performance index; MV DT, mitral

valve deceleration time; NFT, non-flow time; PW, posterior wall; s, in systole; S', systolic peak wave; SV, stroke volume; VTI, velocity time integral. All data were analysed using a one-way ANOVA with a Dunnett's multiple comparison post hoc test with * $p < 0.05$, ** < 0.01, and *** < 0.001 comparing 14 dpi and 35 dpi to Uninfected.
