# Supplementary material for: SARS-CoV-2 infection causes prolonged cardiomyocyte swelling and inhibition of HIF1α translocation in an animal model COVID-19
Source: Front Cardiovasc Med. 2022 Oct 17;9:964512. doi: 10.3389/fcvm.2022.964512 (PMC9618878; doi:10.3389/fcvm.2022.964512)
Supplement: Supplementary file 3 [file Table_2.pdf]

Supplemental Table 2. Antibodies and Fluorescent Staining

Antibody	Dilution	Clone	Catalog No, Distributer
**Laminin**	1/400	Polyclonal	L9393, Sigma-Aldrich
**Topro**	1/1000	-	T3605, Thermofisher
**vWF**	1/1000	Polyclonal	A0082, Dako
**Isolectin B4**	1/50	-	I21411, Invitrogen
**NG2**	1/100	Polyclonal	Ab5320, EMD Millipore
**HIF1α**	1/50	Polyclonal	NB100-479, Biotechne
**WGA**	1/400	-	W32466, Thermofisher

vWF, Von Willebrand Factor, NG2, neuron-glial antigen 2; HIF1α, Hypoxia Inducible Factor; WGA, Wheat Germ Agglutinin.
