# Supplementary material for: SARS-CoV-2 infection causes prolonged cardiomyocyte swelling and inhibition of HIF1α translocation in an animal model COVID-19
Source: Front Cardiovasc Med. 2022 Oct 17;9:964512. doi: 10.3389/fcvm.2022.964512 (PMC9618878; doi:10.3389/fcvm.2022.964512)
Supplement: Supplementary file 4 [file Table_3.pdf]

Supplemental Table 3. Primer sequences used for RT-qPCR

Gene	Primer sequence
HPRT	5’-TGT TGT TGG ATA TGC CCT TG-3’
	5’-ATG GGA CTC CTC ATG TTT GC-3’
VEGFA	5’-TCA CCA AAG CCA GCA CAT AG-3’
	5’-AAA TGC TTT CTC CGC TCT GA-3’
HSP70.1	5’-CAA GTG CAA CGA GGT CCT TT-3’
	5’-CCC CTG GTA GAG TTT GGT GA-3’
HSP70.2	5’-AAC TTT ACC AAG GCG GTC CT-3’
	5’-GGG GTG GAG AAG GAA AAG AG-3’
SLC2A1	5’-ACC AGT TGG AAG CAC TGG AG-3’
	5’-GAG TGT CCG TGT CTT CAG CA-3’
SLC2A4	5’-CTT AGG GCC AGA TGA GAA CG-3’
	5’-TGG AGG TAA CGG GAA GAC AG-3’

HPRT, Hypoxanthine-guanine phosphoribosyl transferase; VEGFA, Vascular endothelial growth factor A; HSP70, Heat Shock Protein 70; SLC2A, Solute Carrier Family 2 Member.
